# Pain in Colorectal Surgery: How Does It Occur and What Tools Do We Have for Treatment?

**DOI:** 10.3390/jcm12216771

**Published:** 2023-10-26

**Authors:** Robert Ivascu, Madalina Dutu, Alina Stanca, Mihai Negutu, Darius Morlova, Costin Dutu, Dan Corneci

**Affiliations:** 1 Department of Anesthesiology and Intensive Care, ‘Carol Davila’ University of Medicine and Pharmacy, 050474 Bucharest, Romania; iulian.ivascu@drd.umfcd.ro (R.I.); dan.corneci@umfcd.ro (D.C.); 2 Central Military Emergency University Hospital “Dr. Carol Davila”, 010825 Bucharest, Romania; 3 Elias University Emergency Hospital, 011461 Bucharest, Romania; 4 Bagdasar Arseni Clinical Emergency Hospital, 041915 Bucharest, Romania

**Keywords:** pain pathways, colorectal surgery, multimodal analgesia

## Abstract

Pain is a complex entity with deleterious effects on the entire organism. Poorly controlled postoperative pain impacts the patient outcome, being associated with increased morbidity, inadequate quality of life and functional recovery. In the current surgical environment with less invasive surgical procedures increasingly being used and a trend towards rapid discharge home after surgery, we need to continuously re-evaluate analgesic strategies. We have performed a narrative review consisting of a description of the acute surgical pain anatomic pathways and the connection between pain and the surgical stress response followed by reviewing methods of multimodal analgesia in colorectal surgery found in recent literature data. We have described various regional analgesia techniques and drugs effective in pain treatment, emphasizing their advantages and concerns. We have also tried to identify present knowledge gaps requiring future research. Our review concludes that surgical pain has peculiarities that make its management complex, implying a consistent, multimodal approach aiming to block both peripheral and central pain pathways.

## 1. Introduction

According to the International Association for the Study of Pain (IASP), pain is ‘An unpleasant sensory and emotional experience associated with actual or potential tissue damage or described in terms of such damage.’ Awareness of the high intensity of intraoperative surgical pain led to the birth of a new medical specialty: anesthesiology (from Ancient Greek roots ἀν- *an*—‘not’, αἴσθησις- *aísthēsis*-‘—‘sensation’, and ‘λογία- *logia*’—‘study’). Underestimation of acute postoperative pain contributes to chronic pain development with a permanently poor quality of life [1]. Surgical pain induces a profound physiologic neurohumoral response, the so-called “adaptive stress response”, which sometimes might exceed its protective role and becomes harmful. Choosing the anesthetic technique and perioperative analgesia equally implies taking into consideration patient factors and surgical procedure stress. Minimally invasive surgical techniques are increasingly utilized, altering analgesic requirements compared with open laparotomy [2].

## 2. Methods: Literature Search

We have conducted a computerized search in different databases (MEDLINE, PubMed, Scopus, Web of Science, and Embase). We have included citations up to December 2022 using the primary search strategy: acute postoperative pain, colorectal surgery, pain assessment, acetaminophen, NSAIDs, ketamine, opioids, epidural anesthesia, peripheral nerve blocks, continuous wound infusion, local infiltration, and their associations. Data has been selected to include consensus conferences, comparative studies, guidelines, multicenter studies, systematic reviews, meta-analysis, large case series, original articles, narrative review articles and randomized controlled trials. Abstracts were screened first, then a full-text assessment of the articles was performed. Case reports were excluded. Finally, a narrative review was carried out.

## 3. Acute Pain after Surgery Pathways

**Figure 1:**
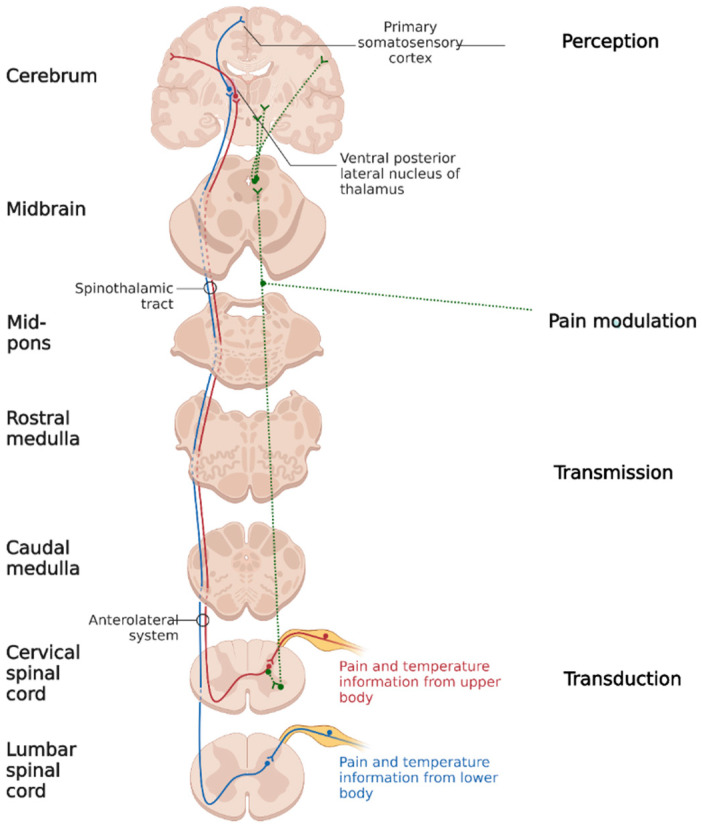
Discriminative pain pathways (created using BioRender.com).

Cortical representation of a pain stimulus implies a four-stage process (see Figure 1):

1. The first stage is **transduction**, meaning a stimulus’ conversion into action potential. Surgical trauma activates nociceptors through their direct mechanical, electrical or thermal stimulation, as well as through cellular destruction, with the secondary release of adenosine, protons, potassium, and bradykinin, which chemically activates the polymodal nociceptors. The antidromic substance P and glutamate released from the afferents of the nociceptive nerve fibers lead to the augmentation of inflammation. All these mechanisms contribute to peripheral nociceptor sensitization, causing allodynia (pain from nonpainful stimuli) and primary hyperalgesia (exaggerated pain from a painful stimulus) [3,4,5].

2. The second step is **transmission**, which is the conduction of the action potential through A δ (somatic parietal pain) and C (visceral pain) nerve fibers. The synapse between primary and secondary neurons takes place in the dorsal horn of the spinal cord. At the synaptic level, the main mediator involved in pain transmission is glutamate, which stimulates receptors coupled with AMPA sodium channels [5]. Repetitively stimulating the secondary neurons increases the membrane density of AMPA receptors. Iterative stimulation leads to the expansion of the nervous afferent territory. Thus, secondary hyperalgesia occurs (amplification of the painful perception of stimulus coming from inside and outside the injured area) through the phenomenon of central sensitization [6,7].

Secondary neuron axons form the ascending tracts: spinothalamic, spinoreticular and spinohypotalamic connected to the hypothalamus, brainstem, limbic system, ascending activating reticulated system and periaqueductal gray substance. These connections take part in the neuroendocrine and vegetative pain response and give the affective/emotional characteristic to surgical pain.

3. The third stage is **pain modulation**. At present, two theories might explain the modulation phenomenon: the first one is based on the descending modulatory system activity (starting from the midbrain periaqueductal gray substance and receiving input from the hypothalamus, thalamus, limbic system, and cortex)—this system is activated by endogenous and exogenous opioids and makes synapses with the inhibitory interneurons from the dorsal horn of the spinal cord. The second one is gate-control theory: a mechanism in the spinal cord in which pain signals go up to the brain and are processed to accentuate or attenuate the possible perceived pain at the spinal cord itself.

4. The fourth stage is pain **perception**. The primary and secondary somatosensory cortex (pain location and duration), limbic system (affective/emotional pain component) and prefrontal cortex (anticipating pain by modeling past experiences) have been identified as regions associated with pain perception [8].

## 4. Pain and Surgical Stress Response

Pain is the main component of surgical stress, leading to the activation of the hypothalamic–pituitary–adrenal axis, with the subsequent increase in serum levels of cortisol, growth hormone and vasopressin, and to sympathetic nervous system activation, all components of the systemic stress response [9]. Cortisol is released very soon after the beginning of surgery with a peak secretion at 4–6 h and, depending on the extent of the surgery, can reach values up to four times higher than the basal ones [10]. High cortisol and glucagon serum levels and decreased insulin secretion caused by sympathetic stimulation lead to catabolic syndrome. Persistent hypercatabolism may lead to increased hospitalization and mortality. Hyperglycemia also increases the infection risk.

Untreated pain causes prolonged sympathetic stimulation which, in association with anemia and perioperative hypoxia, exposes patients with cardiac risk factors to postoperative myocardial injury/infarction (PMI) [11,12]. Data from a large retrospective cohort analysis published in 2020 by Turan et al. concluded that, among patients undergoing noncardiac surgery, high time-weighted average pain scores within 72 h after surgery were significantly associated with greater risk of myocardial injury compared to lower pain scores [13].

Liberal perioperative crystalloid infusion and vasopressin release effects through renin–angiotensin–aldosterone system activation produce interstitial space expansion and postoperative edema. Hypoalbuminemia usually happens due to albumin distribution volume augmentation and increased capillary permeability with secondary escape of serum albumin into the interstitial space. The main consequence is interstitial edema, including at the bowel level, one of the causes of postoperative ileus and postoperative gastrointestinal tract dysfunction with an elevated risk of anastomotic leakage. In addition, manipulation of the bowel, increased sympathetic stimulation, opioid use and fasting are also contributors to the inhibition of gastrointestinal peristalsis [14]. Increased perioperative levels of catecholamine may be associated with pro-metastatic effects [15].

Higher levels of postoperative pain and pain distress can increase morbidity, prevent functional recovery, and reduce the quality of life. Furthermore, suboptimal postoperative analgesia is a risk factor for ongoing opioid use, opioid dependence and persistent post-surgical pain [16].

## 5. Methods to Manage Surgical Pain

The surgical stress response including its physiological derangements becomes a challenge to postoperative patients. The use of standardized enhanced recovery programs in combination with laparoscopic surgery has revolutionized clinical and patient-reported outcomes in elective colorectal surgery. Central to the success of enhanced recovery programs is optimal pain management [17]. Minimally invasive surgical techniques are increasingly utilized, altering analgesic requirements compared with open laparotomy, leading to a decline in the popularity of epidural anesthesia and increasing interest in intrathecal morphine and truncal nerve blocks [2]. There is a paucity of data identifying the ideal analgesic regimen in laparoscopic colorectal surgery and a lack of consensus on the optimal perioperative analgesia strategy in this cohort of patients [17].

The number of hospitalized patients with pre-existing opioid tolerance, chronic pain, or opioid use disorder (OUD) is also increasing, further challenging prescribers and straining healthcare resources [18]. These patients need customized care plans for perioperative analgesia.

Pain management in colorectal surgery requires multimodal analgesia utilizing complementary mechanisms to improve pain control with less high-risk drug exposure. This concept is based on pain prevention and remission through blocking nerve impulse transmission at different stations over the nervous system (as we have shown in the description of pain pathways). Multimodal analgesia has a variety of tools: regional analgesia (peripheral nerve blocks and neuraxial blocks), pharmacological agents (e.g., acetaminophen and anti-inflammatories), neuropathic agents (e.g., gabapentinoids, serotonin reuptake inhibitors, anticonvulsants, corticosteroids, NMDA antagonists, and central alpha-adrenergic agonists), systemic anesthetics (e.g., intravenous lidocaine and inhaled anesthetics), physical therapy, cognitive/behavioral therapies, thermotherapies, and other nonpharmacologic therapies that are beyond the purpose of our review.

## 6. Techniques of Neuraxial and Regional Analgesia

Opioid-related side effects, opioid-sparing anesthesia promoted by enhanced recovery goals, and the opioid abuse epidemic emphasize the need for alternative, opioid-minimizing, multimodal analgesic strategies, including neuraxial (epidural/intrathecal) techniques, truncal nerve blocks, and lidocaine infusions [2].

### 6.1. Neuraxial Techniques

The spinal cord is central to the transmission of pain. Local anesthetic injection into the neuraxial space segmentally blocks nerve transmission of nociception to the brain [2]. Published two decades ago, the largest multicenter randomized control trial investigating the role of epidural analgesia in major abdominal surgery reported reductions in pain scores and respiratory complications in the epidural analgesia group, without additional morbidity or mortality benefits [19]. Since then, several systematic reviews provided consistent findings on the reduction in rest pain after major abdominal surgery, and non-conclusive evidence for a positive impact on dynamic pain [20,21,22]. 

According to ERAS protocols, thoracic T7-T10 level epidural anesthesia (TEA) is the gold standard for obtaining adequate analgesia in open colorectal surgery [23]. In a study published in 2017, TEA was at that point the most commonly used regional technique among ERAS protocols in 15 different healthcare facilities mostly located in North America and 1 in New Zealand [24]. Its use has proven advantages: prevention of intestinal mucosa degradation, metabolic status improvement by minimizing surgical stress, and mitigation of the postoperative ileus. Still, no mortality benefit associated with epidural analgesia use has been reported in most of the systematic reviews conducted. A large cohort study using the US National Surgical Quality Improvement Program (NSQIP) database (64,119 patients having received neuraxial or regional anesthesia) revealed significantly lower odds of several postoperative complications, especially respiratory complications, and decreased hospital length of stay, but not mortality [25]. 

The popularity of neuraxial techniques appears to be declining worldwide [26]. Possible technical complications (e.g., dislodgement, leakage, occlusion, nerve injury, and paraplegia) with consecutive analgesia failure and hemodynamic compromise requiring additional intravenous fluid therapy and vasopressor support, especially in elderly patients, might be reasons for epidural use restraint in colorectal surgery. 

TEA does not exclude a multimodal approach, for example, in combination with intravenous acetaminophen. It seems to provide superior postoperative pain management compared with TEA alone [27].

What about TEA used in laparoscopic colorectal surgery? It is believed that the laparoscopic technique is less painful than the open technique, but that depends on how pain is managed. Results of a prospective cohort study assessing postoperative pain on 434 patients after elective colorectal surgery within the ERAS perioperative program concluded that patients undergoing minimally invasive colonic surgery experienced more pain on the day of surgery and less pain on postoperative days 2 and 3 vs. open colonic surgery; thus, patients having open colonic surgery would probably benefit from epidural analgesia for 3 days instead of 1 [28]. 

A multicenter, prospective, observational cohort study of variation in practice in perioperative analgesia strategies in elective laparoscopic colorectal surgery (the LapCoGesic study) released in 2020 has demonstrated a steady movement away from the use of epidural analgesia in recent times, with a trend towards the use of patient-controlled analgesia and spinal diamorphine in elective laparoscopic colorectal surgery [17]. Although epidural analgesia in laparoscopic colorectal surgery reduces early postoperative pain (24 h) [29], there are conflicting findings on side effects, time to return of bowel function, and hospital length of stay. A higher overall complication rate has been reported [30]. 

However, is TEA far superior to other regional anesthesia techniques? Analgesia obtained after TEA is superior to that obtained by continuous wound infiltration in laparoscopic surgery [31], but no difference has been noticed when comparing TEA to transversus abdominis plane (TAP) block [32]. 

### 6.2. Intrathecal Analgesia

Intrathecal morphine has a prolonged duration of action and higher potency. A clinically significant opioid-sparing effect on the first postoperative day has been consistently reported. Concerns regarding delayed respiratory depression have limited the widespread use of intrathecal morphine [33]. The recommended postoperative monitoring for intrathecal morphine is the same as that for patient-controlled analgesia of opiates. Comparing intrathecal morphine or diamorphine to intrathecal local anesthetic, or to systemic analgesia in laparoscopic colorectal surgery, lower pain scores and opioid consumption up to the third postoperative day in the intrathecal opioid group have been reported. Nausea similarly has affected both cohorts, and pruritus has disproportionately impacted the intrathecal opioid group [34,35,36]. Studies comparing intrathecal morphine with epidural analgesia reported a reduction in the incidence of hypotension and hospital length of stay with the use of intrathecal morphine; postoperative pain, limited to the first 24 h after surgery, was lower in those patients who received epidural analgesia [2]. Hydromorphine, buprenorphine, and diamorphine are alternative hydrophilic opioids that can be administered via the neuraxial route for pain management [37].

### 6.3. Transversus Abdominis Plane Block (TAP)

The context of the increasing prevalence of minimally invasive surgery and declining popularity of neuraxial techniques makes truncal blocks, and especially TAP, important analgesic tools in colorectal surgery. Viderman et al. performed a meta-analysis on TAP used in colorectal surgery, including 8 RCTs published before September 2021 involving 615 patients. The need for opioids and the intensity of pain at rest within 24 h after laparoscopic and combined (laparoscopic and open) surgeries were significantly lower in the TAP block group compared with the “no block” group; however, the effect of TAP block in reducing opioid requirements was more pronounced in the combined group (laparoscopic and open surgery). TAP block did not influence postoperative nausea and vomiting and length of hospital stay after laparoscopic and combined surgeries [38]. A recent review recommends subcostal transversus abdominis plane blocks as a valid alternative for patients undergoing laparotomy in whom neuraxial blocks are contraindicated [39]. Epidural analgesia was compared with TAP in colorectal surgery in a systematic review published in 2021 including six randomized control trials. Functional recovery in the TAP group was found to be superior to epidural analgesia with a reduction in total opioid consumption, time to ambulate, urinary catheter time, and postoperative hypotension in the context of comparable pain control [40].

### 6.4. Rectus Sheath Block (RSB)

Rectus sheath block (RSB) is an anesthetic technique first described in 1899 but only recently used. Literature data have shown the effectiveness of this technique, either by using a single shot or catheter technique (RSC). In 2019, a study published by Uvarov in major abdominal surgery proved that RSB analgesia is superior to the systemic one [41]. Compared to TEA, the rectus sheath catheter technique has proved the same degree of analgesia, but with fewer side effects [42,43]. RSB has not proved its usefulness in laparoscopic abdominal surgery [44].

### 6.5. Continuous Wound Catheter

Pain caused by peritoneum injury has been addressed by trying to block pain through a preperitoneal catheter with a local anesthetic. A meta-analysis performed in 2019 on 564 patients with colorectal resection has shown a decrease in the need for opioids when using a continuous wound catheter, although with moderate evidence [45]. Intra- and postoperative analgesia obtained with continuous wound catheter use seems to be inferior to that obtained using other regional techniques of analgesia. Mann et al. suggest using continuous wound catheter analgesia in emergency laparotomy as part of the multimodal analgesia [46]. 

A comparison between techniques of regional analgesia in open and laparoscopic/robotic surgery can be found in Table 1. 

### 6.6. Lidocaine

The analgesic effect of lidocaine consists of sodium channel blockade at the neuronal level. Intravenous lidocaine produces prolonged analgesic effects with reduced inflammation, neuropathic pain, and hyperalgesia and may positively affect wound healing and cognitive function [47,48]. In addition to reducing the painful stimulus and the need for opioids, lidocaine also has a direct effect on intestinal motility, diminishing paralytic ileus [49]. Meta-analysis by Cooke et al. has shown that intravenous lidocaine administration in colorectal surgery is associated with reduced pain scores, ileus duration and hospitalization when compared with a placebo [50]. Currently, the optimal dose and duration of lidocaine infusion are still debated, with the most commonly recommended protocol being a bolus of 1.5 mg/kg at induction, followed by a continuous intraoperative infusion of 2 mg/kg/h, with optional continuation in the postoperative period up to 48 h [51]. The ALLEGRO (rAndomised trial of intravenous Lidocaine in acceLErating Gastrointestinal Recovery after cOlorectal surgery) trial (ISRCTN52352431) is on course, assessing the benefit of perioperative intravenous lidocaine in improving the return of gastrointestinal function after elective minimally invasive (laparoscopic or robotic) colorectal surgery [52]. Intravenous lidocaine may also have antineoplastic effects, as shown by a large prospective study (e.g., NCT 04316013) investigating its impact on cancer [53]. ERAS guidelines recommend IV lidocaine use as part of multimodal analgesia in colorectal surgery, representing an alternative to thoracic epidural analgesia in laparoscopic surgery.

## 7. Techniques of Parenteral Analgesia

### 7.1. Opioid Analgesia

Acting on central and peripheral μ, κ and δ receptors, opioids modulate the release of nociceptive neurotransmitters (substance P or glutamate) and hyperpolarize the neuronal membrane, reducing neuronal excitability. Although they confer strong and effective analgesia, their side effects (e.g., drowsiness, respiratory depression, urinary retention, ileus, constipation, nausea, and pruritus) can limit optimal analgesic dosing and may lead to poorer analgesia and recovery. Despite opioid avoidance in the colorectal surgery ERAS goal, opioids are sometimes the mainstay of drug treatment for analgesia in the perioperative period.

Empiric opioid regimens should include consideration of optimal agent selection, dosing, route of administration, and supportive therapies [18]. For patients without prior exposure to opioids, there are “generally preferred” agents with less genetic variability in response, decreased reliance on end-organ function for safety, fewer drug–drug interactions and lower histamine release, such as oxycodone, hydrocodone, and hydromorphone instead of morphine, codeine, and tramadol [54,55]. In patients with a prior opioid exposure history, agent selection should always be tailored and monitored for adjustment [18]. Patients with opioid tolerance, chronic pain, and/or opioid use disorder require higher opioid doses and more supportive therapies.

The intravenous (IV) route of administration is the fastest route to provide analgesia. Intermittent IV bolus doses as needed are suggested for titration of opioids for severe acute pain, but IV analgesic regimens should generally be converted to enteral ones as soon as appropriate [56]. In patients requiring prolonged analgesia, it is suitable to administer opioids through a patient-controlled analgesia pump (PCA) without basal infusion rate setting and with continuous monitoring of patient vital signs [51]. Pain regimens should be evaluated and adjusted at least daily through multidimensional pain assessments to optimize efficacy and safety endpoints [18]. Higher doses of opioids confer the risk of longer-term dependence, with up to 10% of opioid-naïve patients continuing opioid use beyond 90 days after a range of major and minor surgeries, with a substantial burden of morbidity and cost to the community [2]. 

The percentage of hospitalized patients on chronic medication for opioid use disorder (MOUD) is increasing, and hospital providers must therefore understand the fundamental concepts relating to managing pain in this high-risk population [57] Published evidence and experience support the continuation of methadone (mu-opioid receptor agonist and NMDA antagonist) and buprenorphine (partial mu-opioid receptor agonist and kappa-opioid receptor antagonist) regimens during acute painful episodes, including through surgical encounters. Their continuation improves pain control, reduces the risk of relapse, and has been shown to decrease as-needed opioid requirements in this patient population [18].

Exogenous opioids exert innate and acquired immunity inhibition, adding to immunosuppression generated by surgical stress and pain [58,59]. As a result, their use could increase the risk of postoperative infections and neoplastic cell dissemination. Enhanced tumor growth, angiogenesis, and distant spread owing to transactivation of vascular endothelial growth factor (VEGF) receptors and increased expression of opioid receptors suggest a plausible link between opioid prescription and cancer outcomes [60]. Their immunosuppressive effects depend on dose and type. An opioid with lower activity at the level of specific receptors (such as tramadol) is associated with a lower degree of immunosuppression [58].

In the context of a worldwide opioid epidemic, strategies to minimize opioid use in the perioperative setting, whilst optimizing patient comfort and functional recovery, are priorities for multimodal analgesia.

### 7.2. Non-Opioid Adjuvant Analgesia

**Non-steroidal anti-inflammatories (NSAIDs)** exert their analgesic and anti-inflammatory effects by inhibiting cyclooxygenase with a consequent decrease in the synthesis of prostaglandins in injured tissue. Together with acetaminophen, they are the cornerstone of multimodal analgesia in major surgery, decreasing the total opioid use and reducing the duration of postoperative ileus. Although several observational studies have shown that the administration of NSAIDs, especially non-selective, is associated with a higher risk of anastomosis leak in colorectal surgery, there is still not enough strong evidence to restrict their use [61,62,63]. However, caution is suggested for patients experiencing digestive anastomosis.

**Acetaminophen.** A centrally acting analgesic, paracetamol, decreases total opioid use and has a synergistic effect when administered with an NSAID [64] Unlike NSAIDs, it does not interfere with platelet aggregation.

**Nefopam.** Like paracetamol, nefopam is an analgesic with central action. It does not interfere with platelet aggregation, but through its sympathomimetic activity, it can increase the risk of arrhythmias. Combined with paracetamol or NSAIDs, it has a synergistic effect, managing to decrease opioid use and provide effective analgesia in major surgery [65,66].

**Metamizole.** Despite its excellent analgesic properties, metamizole is less used as a result of the associated risk of agranulocytosis, although its incidence remains very low [67,68]. Taken together with NSAIDs or opioids, it potentiates their analgesic effect while decreasing the total opioid use. In the absence of adverse gastrointestinal and renal effects of NSAIDs, metamizole use in postoperative multimodal analgesia could spare NSAID use in patients in whom they are contraindicated [69].

**NMDA receptor antagonists** (e.g., ketamine and magnesium sulfate) have an analgesic effect, diminishing central pain sensitization and preventing the occurrence of postsurgical chronic pain [70] Administered in sub-dissociative doses, ketamine contributes to better pain control and decreased opioid requirements [71], especially in patients with high opioid tolerance. Although the maximum analgesic benefit appears to be in upper abdominal, thoracic and orthopedic major surgery [72], it can also be used with good results in colorectal surgery. Thus, in addition to its analgesic and opioid-sparing effects, ketamine administration led to a decrease in the incidence of nausea and vomiting, fewer areas of hyperalgesia and less residual pain in the first six postoperative months [73]. A recent meta-analysis confirms the beneficial effect of intraoperative magnesium sulphate administration, with decreased opioid requirements in the first 24 h postoperatively and better pain control in major abdominal surgery [74,75].

**Gabapentin.** By inhibiting neuronal presynaptic calcium influx, gabapentin decreases neuronal excitability, contributing to better control of perioperative pain with a reduction in the need for opioids and possibly preventing chronic surgical pain occurrence [76]. Considering their side effects (e.g., sedation, dizziness, headache, visual disturbances, and edema), it is recommended to limit their administration in major surgery to patients who are already in chronic treatment with gabapentin, with chronic pain or a high tolerance to opioids [51]. Thus, most guidelines, including the ERAS guide for colorectal surgery, propose the administration of a single preoperative dose of gabapentin together with paracetamol and NSAIDs [23,51].

**α2 receptor agonists.** Analgesic properties of α2 receptor agonists (clonidine, dexmedetomidine) are due to mechanisms that are not yet fully elucidated. Widely used for their sedative, anxiolytic and sympatholytic effects, they are still underused as adjuvants in multimodal analgesia, despite large amounts of evidence in the literature indicating that they decrease the need for anesthetics and opioids in both loco-regional and systemic administration [77,78]. In abdominal surgery, clonidine intravenous or epidural use decreases, in equivalent doses, the need for postoperative analgesia, with similar side effects. The benefits are also maintained with dexmedetomidine intravenous administration in colorectal surgery [79,80]

**Corticosteroids.** Intravenous administration of preoperative high-dose corticosteroids (dexamethasone dose above 0.1 mg/kg or methylprednisolone 30 mg/kg) reduces postoperative pain and the duration of hospitalization in colorectal surgery without increasing complication incidence, including anastomotic leak risk [81,82,83].

Are there any knowledge gaps in pain management in colorectal surgery?

The expansion of minimally invasive surgery leads to adaptation of the anesthetic and analgesic requirements compared to those required for open surgery.

Epidural analgesia continues to be recommended by ERAS protocols for major abdominal surgery despite its significant side effects, waning popularity, and the increasing use of minimally invasive surgery.

Intrathecal opioids are an attractive option for perioperative analgesia given the ease of delivery, high success rate during instrumentation, early intraoperative and postoperative opioid reduction, and effective postoperative pain management.

There is a need for large multicenter randomized trials directly comparing epidural analgesia with intrathecal morphine in major abdominal (open or laparoscopic) surgery. Intravenous lidocaine’s potential anti-inflammatory, analgesic, and antineoplastic benefits must be researched further.

There is a need for large-scale comparative studies on multimodal analgesic protocols designed specifically for laparoscopic, robotic-assisted, and open surgery, respectively.

## 8. Conclusions

Perioperative pain management has undergone a change in thinking over the past few decades. If traditionally opioids were the mainstay of drug treatment for analgesia, nowadays, multimodal analgesia is the right choice. This involves the simultaneous use of pharmacological and non-pharmacological analgesia techniques blocking pain pathways at distinct levels. Through their synergistic effects, they ensure better pain control and lower opioid consumption, thus reducing the associated side effects. As a result, opioid-free multimodal analgesia or sparing techniques occupy a key place in protocols for enhanced recovery following colorectal surgery.

## Figures and Tables

**Table 1:** Comparison between techniques of regional analgesia.

Analgesic Techniques and Surgery Type	Benefits	Risks
**Epidural analgesia in open colorectal surgery**	Reduced respiratory complications. Reduced rest pain scores [19]. Prevents intestinal mucosa degradation. Reduced time to return of bowel function. Reduces surgical stress [24]. Decreased hospital length of stay [25]. ERAS guidelines recommendation [23].	No morbidity or mortality benefit [19]. Increase analgesia failure [2]. Increase hypotension requiring treatment [2]. Technical and neurological complications.
**Epidural analgesia in laparoscopic/robotic colorectal surgery**	Reduced pain (24 h) [29]. Conflicting findings on side effects, time to return of bowel function, and hospital length of stay [30]. Not superior to TAP [32].	Higher overall complication rate [30].
**Intrathecal opioids in open/laparoscopic/robotic surgery**	Reduced opioid requirement (24 h) Reduced rest pain (24 h) and dynamic pain (up to 48 h) Reduction in hypotension [34].	Dose-dependent delayed respiratory depression [33]. Dose-dependent pruritus and nausea. Sedation similar to systemic opioids [35,36].
**Transversus abdominis plane block in open/laparoscopic/robotic surgery**	Reduced opioids need Improved early pain control [38]. Compared to epidural: - Superior functional recovery - Reduced time to ambulate, - Reduced urinary catheter time. - Reduced hypotension [40]. Easy to perform and cost-effective.	Did not influence postoperative nausea and vomiting and length of hospital stay.
**Rectus sheath block in open surgery**	Compared to epidural: - Comparable degree of analgesia - Fewer side effects [42,43].	Not useful in laparoscopic surgery [44].

## Data Availability

Not applicable.
